# Dementia-related stigma in physicians: a scoping review of stigma-reduction interventions

**DOI:** 10.3389/frdem.2025.1601462

**Published:** 2025-07-22

**Authors:** Alison Warren, Zan Wynia

**Affiliations:** ^1^The Department of Clinical Research and Leadership, School of Medicine and Health Sciences, George Washington University, Washington, DC, United States; ^2^The Frame-Corr Laboratory, The George Washington University School of Medicine and Health Sciences, Washington, DC, United States; ^3^Harvard University Extension School, Division of Continuing Education, Cambridge, MA, United States; ^4^Anschutz Medical Campus General Internal Medicine, University of Colorado, Aurora, CO, United States

**Keywords:** stigma, bias, dementia, Alzheimer’s disease, intervention, physician, healthcare personnel

## Abstract

**Introduction:**

Despite progress in dementia diagnosis and treatment, physician-held stigma remains a significant barrier to early recognition and effective care. Stigmatizing attitudes among healthcare professionals can negatively impact diagnosis rates, clinical interactions, and care quality for people living with dementia.

**Methods:**

This scoping review was conducted following Arksey and O’Malley’s framework. Peer-reviewed literature from 2014 to 2024 was systematically reviewed to identify and evaluate interventions aimed at reducing dementia-related stigma among physicians. A total of 14 studies met inclusion criteria, examining educational, skill-building, and person-centered approaches.

**Results:**

Interventions included brief workshops, online modular training, and interdisciplinary methods integrating person-centered frameworks and behavior management tools. Validated outcome measures used in the studies included the Alzheimer’s Disease Knowledge Scale (ADKS), the Dementia Negative Stereotype Scale (DNS), and the General Practitioners Confidence and Attitude Scale for Dementia (GPACS-D). Across studies, interventions were found to improve clinical confidence, reduce negative stereotypes, and enhance care quality.

**Discussion:**

Findings highlight the importance of culturally sensitive and interdisciplinary interventions to address stigma, improve clinical confidence, and enhance care quality, particularly in low-resource settings. Notable gaps remain in understanding the long-term impact and scalability of such interventions. This review aims to contribute a deeper understanding of the barriers and facilitators to implementing dementia care practices, offering a conceptualization for enhanced physician education and improved health outcomes for persons with dementia. We offer recommendations for future research to develop tailored strategies that support stigma reduction and improve care delivery.

## 1 Introduction

Over 55 million people worldwide are currently living with dementia, with a projected doubling of this number over the next 20 years (2024 Alzheimer’s disease facts and figures, 2024; Alzheimer’s Disease International, 2024). As such, there is a societal need to overcome barriers to early recognition and intervention, as well as person-centered treatment for patients and caregivers. Based on the works of Rogers (1961) and Kitwood (1993), person-centered care prioritizes the person, rather than the disease, and values the person’s unique identity, preferences, and needs, recognizing their personhood regardless of cognitive decline (Mitchell and Agnelli, 2015). For example, offering a person with dementia choices about their meals and respecting their routines fosters dignity and wellbeing. This approach places the person at the center of their own care and helps to preserve the identity and autonomy of people with dementia, improves psychological outcomes, and strengthens trust between patients and caregivers by emphasizing respect, empathy, and partnership in care (Mitchell and Agnelli, 2015). Stigma, including implicit biases and beliefs that affect attitudes and behavior (Auerbach et al., 2018), is a major barrier in this regard (Herrmann et al., 2018). Stigma encompasses both the active process of labeling, stereotyping, and discrimination within power dynamics (Hatzenbuehler et al., 2021) and its consequential devaluation and dehumanization of individuals through discrediting perceptions, attitudes, and behaviors that ultimately lead to a “spoiled identity” (Goffman, 1974; Sabat, 2006). “A “spoiled identity” refers to a social identity that has been discredited or devalued due to a stigmatized trait, causing the individual to be seen as flawed thus socially discounted (Bacsu et al., 2024; de Medeiros and Girling, 2021; Goffman, 1974). The spoiled identity perception positions the person with dementia as merely a passive, dependent care recipient (de Medeiros and Girling, 2021), rather than one who can actively and meaningfully participate in care plans. Stigma is a complex construct that can occur across several contexts, such as self-stigma (internalization of negative stereotypes), interpersonal, family and caregiver, cultural and societal, and institutional (Rosin et al., 2020). Famed dementia researcher Tom Kitwood used the broader term, “malignant social psychology,” to refer to the dysfunctional, yet often unintentional behaviors that depersonalize persons diagnosed with dementia (Kitwood, 1993; Warren, 2023a). They include a broad range of behaviors that negatively affect persons with dementia, including stigmatization, as well as outpacing, labeling, exclusion, and invalidation (Kitwood, 1993).

Physicians play a pivotal role in dementia care, yet stigma among physicians tends to manifest as discomfort, avoidance, nihilistic attitudes (nothing can be done, hopeless and/or a burden on the system), and misconceptions that may negatively affect patient care and outcomes (Bacsu et al., 2020; Beaulieu et al., 2017). For example, a recent World Alzheimer’s Report (2024) showed that 64% of healthcare providers believe that dementia is a normal part of aging (Alzheimer’s Disease International, 2024). Stigmatizing attitudes that are specific to dementia include conceptualizations that persons living with dementia are akin to the “living dead,” that they will inevitably become incompetent and burdensome with little to no quality of life, and that they cannot contribute to society (Rosin et al., 2020).

Critically, dementia-related stigma in healthcare contributes to significant barriers in care practices, including timely diagnosis and treatment (Bacsu et al., 2020), and underutilization of health and social services (Bacsu et al., 2022). Further, recent evidence suggests that dementia-related stigma may be higher in healthcare providers than in the general public (Herrmann et al., 2018; Lock et al., 2023). In fact, physician attitudes towards dementia are a stronger key determinant than medical knowledge of dementia of whether a patient receives a full clinical dementia assessment (Mason et al., 2020). Yet, there is a paucity of research focusing on destigmatizing interventions targeted towards physicians (Bacsu et al., 2020).

Dementia-related stigma is derived from implicit bias (Auerbach et al., 2018), lack of confidence (e.g., diagnosis disclosure) (Cartz-Piver et al., 2023) lack of education, negative social constructions, and sociocultural influences (Avari and Meyers, 2018). In patients experiencing or at risk for cognitive decline, early detection is a critical determinant of disease management outcomes (Thyrian et al., 2016), but the stigma associated with the diagnosis of dementia contributes to physicians’ reluctance to diagnose patients presenting with symptoms (Cartz-Piver et al., 2023). Additionally, difficult symptoms such as the behavioral and psychological symptoms of dementia (BPSD) can further compound this issue. Addressing the multiple factors that may foster stigma are critical to improving early detection and quality care management, including physician knowledge, competence, confidence, and attitudes (Crombie et al., 2024), as well as adequate experiential contact with persons with dementia (Goldman and Trommer, 2019). The multidirectional interactions between one’s level of dementia-specific knowledge, psychological schemas, and sociocultural milieu lend to its complexity, which is transdisciplinary in nature and extends to the emotional, ethical, and practical dimensions that emphasize the need for comprehensive, multifaceted, and transdisciplinary interventions (Repko and Szostak, 2017).

## 2 Research question

The aim of this scoping review was to answer the following main research questions:

1. *“What interventions have been developed to reduce stigma and improve physicians’ attitudes, competence, confidence, and practices in the care of individuals with dementia?” What are the key components of those interventions?*

## 3 Methods

A scoping review was selected to answer this research question. Following Arksey and O’Malley’s (2005) framework for scoping reviews (identifying the research question; identifying relevant studies; study selection; charting the data; collating, summarizing and reporting the results), we aim(ed) to review the extant literature, including key stakeholder groups to include a broad range of perspectives. A scoping review was chosen to answer this research in line with recommendations from Munn et al. (2018) as we aim to identify key concepts and factors in dementia-stigma reducing interventions for physicians, identify current gaps in our knowledge on this topic, and examine current research approaches.

### 3.1 Search strategy

In October 2024, the available peer-reviewed literature was examined to identify what is known about dementia-related stigma interventions in physicians, including biases, beliefs, and attitudes about dementia. With the aid of a George Washington University School of Medicine and Health Sciences librarian, appropriate key words and search strings were identified and utilized. The search included PubMed, Scopus, CINAHL, and PyschoINFO. Studies were included if they were published in a peer reviewed journal and which evaluated an intervention designed to improve dementia related stigma, attitudes, and/or bias among physicians or among health care workers generally but which included physicians in their sample. Studies were excluded if the intervention involved biomedical education only without the elements of attitude assessment and intervention, as increasing knowledge alone has suggested to be ineffective (Rosin et al., 2020). However, it’s important to note that we did not exclude interventions that included biological education alongside stigma education or mention of recent biological advances in our understanding of dementia and dementia care. Since the focus of this study was specific to stigma in physicians toward persons with dementia, studies that involved students, nurses, other allied health professionals, and staff were excluded, but were included if these populations were present alongside physician populations. Additionally, the search was restricted to studies published between 2014 and 2024, fully available in English, and the full text freely available through library services at George Washington University or Harvard University.

### 3.2 Data extraction

Titles/abstracts were initially screened for inclusion in the final sample by the two authors of this paper (AW and ZW). Articles’ full text were then reviewed by AW and ZW for final inclusion. Once all included studies had been identified, ZW and AW used a standardized template in Excel to extract the following information: authors, originating discipline, stakeholder representation, publication title, year of publication, study location, study population, intervention type/description, duration of intervention, an overview of methodology, outcome measures, and results/conclusions. If disagreements occurred, a 3^rd^ impartial reviewer was to be brought in to make a final decision.

### 3.3 Evidence summary

In line with the primary research question, the specific components of the question we want to answer through this scoping review are: (1) What types of interventions are used to address dementia-related stigma in physicians, (2) what are the key components of those interventions, (3) What outcome measures are employed to capture the multiple facets involved in stigma, (4) what are the knowledge gaps, barriers, and facilitators of dementia-related stigma in physicians. These questions served to structure and inform our analysis by categorizing stigma-reduction interventions into specific approaches (e.g., educational, hybrid, etc.) and to further link the measurements and outcomes (Table 1).

**Table 1:** Study characteristics.

Author (Year)	Location	Intervention type/duration	Study population	Methodological overview	Results
Albrecht et al. (2022)	Wisconsin (online intervention)	Workshop focused on the DICE (describe, investigate, create and evaluate) approach to dementia care. Prior to the COVID-19 pandemic, three annual one-day in person comprehensive trainings were held. In person training included didactics, problem solving with case examples, and Q & A sessions. Participants were encouraged to share with each other their personal experiences of working with clients with BSPD. All participants were given the DICE manual as well. During COVID-19, training was moved online. In the online format pre-recorded modules replaced the in person didactics, with modules covering the same topics. Online modules also contained “e-simulations” to test participant learning in two case-based simulations. The average time to complete the online training was 3 h.	122 health care providers, 3 of whom were physicians	The DICE Approach (DICE) is a training tool for managing BPSD from a person-centered perspective. DICE training was given in person and online and case consultations were offered for challenging situations.	Participants demonstrated significant improvement from pre to post DICE training in knowledge, attitudes, and self-efficacy. The authors summarize that DICE training improved the ability for professionals at various levels to manage BPSD and support caregivers in doing so as well.
Arsenault-Lapierre et al. (2022)	Canada (Primary care clinics)	Audits were performed at 8 primary care sites that had implemented innovative primary care dementia models that included a clinician education component. Four sites implemented a embedded-assessor model, two had implemented a collobrative memory clinic model, and two had implemented a hybrid of those approaches.	Eight clinics received audits on dementia care	Eight sites that had dementia care models received one audit and feedback cycle. “Audit consisted of (a) chart review to assess quality of dementia care indicators, (b) questionnaire to assess the physicians’ knowledge, attitudes and practice toward dementia care, and (c) semi-structured interviews to understand barriers and facilitators to implementing these models.” Feedback presentations were given to clinicians and staff. Finally, discussions insights and proposed solutions were carried out.	Insights regarding organizational factors and clinical competency and attitudes were discussed, along with potential solutions for improvement.
Bentley et al. (2019)	Australia	Interactive online educational course that contained 3 h of content tailored to IMGs. Focus areas, each with their own module, included (1) recognizing dementia in general practice, (2) diagnosing dementia in general practice, (3) dementia progression, and (4) managing dementia in general practice. Each module consisted of a video, assessment questions, and additional resources for further learning. Videos had a conversational format containing GPs, nurses, caretakers, and people living with dementia. Prior to starting the program participants participated in semi-structured interviews and completed 3-h of reading.	33 international medical graduates and nurses	4 online modules delivered over 3 h to improve dementia detection, diagnosis, and management. Pre/post assessment of knowledge, confidence, and attitudes.	Improvements observed in dementia awareness, knowledge, confidence, and attitudes, with evidence of changes in clinical behavior. Authors highlight value of a systematic framework to increase awareness and detection of dementia in PCPs which could extend to colleagues.
Cartz-Piver et al. (2023)	Universities in France, Bulgaria, & Poland	In person workshop that varied in length between study sites, but which were the content of which were governed by the same core learning objectives. The learning objectives were to teach the “why” and “how” of managing dementia through ethical and practical content, and avoiding “what” with mainly academic content. Content also contained a common set of questionnaires and tools, including an auto-questionnaire about socio-demographic characteristics and the Dementia Negative Stereotypes Scale (DNS) and the Dementia Clinical Confidence Scale.	134 GPs and 58 medical trainees	The Antistigma education intervention teaches “Why” and “How” to manage dementia (rather than only the “What”). To measure stigma, The Dementia Negative Stereotype Scale (DNS) and The Dementia Clinical Confidence Scale (D-CO) were given pre and post training.	Post training intervention, negative stereotypes improved, stigma was reduced, and confidence improved. “Participants who benefited best from the Antistigma education intervention were those without training in Geriatrics and those working in nursing homes (who reduced the most D-NS), as well younger participants and those who managed less than five people living with dementia per week (who increased the most D-CO).” While not stated, this is consistent with the literature that those who are younger and have more patient exposure tend to have less stigma.
Crombie et al. (2024)	Australia (family medicine practice groups)	Intervention delivered in four 2-h sessions (8 h total) by a geriatrician and psychogeriatrician, both experts in dementia. Topics covered were (1) recognizing dementia in general practice, (2) risk factors and both pharmacological and non-pharmacological management, (3) BSPD and depression, (4) carer stress, (5) support services and respite, (6) legal issues and end of life issues, (7) advanced directives and palliative care.	14–16 GPs	Two-stage, mixed methods design, Stage 1: 16 GPs participated in semi-structured interviews. Stage 2: 14 (different) GPs - pilot educational intervention delivered by a geriatrician and psychogeriatrician, plus pre/post surveys, and post-training interviews.	Stage 1 analysis revealed 3 themes regarding dementia management, “(1) attitudes to and experiences of dementia; (2) supporting people living with dementia; and (3) knowledge, education and training of dementia.” Stage 2 intervention was shown to improve attitudes, knowledge, and confidence in GPs, who demonstrated nihilism and lack of confidence pre-intervention.
Edwards et al. (2015)	United Kingdom (primary care clinics)	A one-hour in person workshop that was initially piloted and refined via clinician feedback. Intervention primarily consisted of a PowerPoint presentation and a printed handbook of slides with additional case examples. Presentation consisted of 3 focus areas, (1) introduction to dementia and its subtypes, (2) introduction to person-centered dementia care, and (3) summary and consolidation of earlier sections. Final intervention after pilot feedback consisted of the PowerPoint, printed handbook with slides and four case examples, and a training manual with detailed guidelines for delivery.	94 clinic staff, 30 of whom were physicians	Knowledge and attitudes about dementia were measured pre- and post-educational intervention to 94 practice staff, and GPs were compared non-GPs.	Significant improvements were observed post-intervention in understanding of person-centered dementia care, as well as dementia attitudes, awareness, knowledge, and recognition. Authors indicate that educational interventions that emphasize person-centered care are beneficial in primary care practices.
Mason et al. (2020)	Australia	“Recognizing, Diagnosing, and Managing Dementia in General Practice Workshop” consisted one 3-h in-person workshop delivered by medical educators. Two primary focus areas were (1) recognizing and diagnosing dementia and (2) managing dementia in clinical practice. In particular there was a strong emphasis on the lived experience of people living with dementia to help GPs consider diagnosis and management through a biopsychosocial lens.	446 GPs and medical trainees	In-person dementia education workshop. The intervention focused on properly identifying, diagnosing, and managing dementia in general practice.	A significant increase in scores was observed post-intervention. The authors highlight the value of targeted interventions to improve attitudes and confidence in GPs.
Perales-Puchalt et al. (2022)	Kansas (remote intervention)	The Dementia Update Course was a 6.5 h hybrid intervention held 5 times over a 1-year period, except for one course which was condensed to a 4-h format. The course foundations were constructed from the Health Belief Model and Social Learning Theory. Content included clinically focused lectures, case examples with discussion, videos, and material for additional reference. Topics included dementia detection and diagnosis, treatment, care for cognitive and behavioral symptoms, and how to integrate tools into the healthcare workers’ daily workflow. There was also content on patient empowerment following the Clinical Empowerment Model and cultural competence. Attendees also received copies of recommended cognitive screening tools and course materials.	22 PCPs and 32 health navigators	The intervention was a dementia update, including several topics. Pre-post training assessments included participant satisfaction, competency, ADRD-related attitudes, and the General Practitioners Confidence and Attitude Scale for Dementia (GPACS-D).	PCPs demonstrated improvements in outcomes post-training. The authors conclude that this brief training improved dementia care competency in PCPs.
Pond et al. (2018)	Australia	Education academic detailing session led by a trained peer or medical educator. Sessions focused on (1) instruction on use of the General Practitioner Assessment of Cognition scale, (2) interactive presentation on dementia diagnosis, management, and workup as based on RACGP dementia guidelines, (3) exploration of GP perceived barriers to dementia diagnosis, and (4) business case outlining cost recovery potential of dementia assessment. At conclusion GPs were provided with printed copy of RACGP guidelines and a summary poster.	168 GPs	The practice-based academic detailing intervention included training of assessment, screening, diagnosis, and management of dementia, along with education about GPs barriers to diagnosis and cost-effective assessment approaches.	No significant effect on primary outcome measures, but secondary outcome measures improved post intervention. “Practice-based academic detailing did not improve patient quality of life or depression scores but did improve detection of dementia in primary care and patient satisfaction with GP communication”
Sá Mayoral et al. (2021)	Brazil (Primary care clinics)	6-h of lectures delivered by a geriatrician with experience in dementia care. Lecture topics covered dementia definition, the epidemiology of dementia, and the diagnosis and management of dementia symptoms.	34 GPs	6 dementia lectures lasting about 6 h total were presented to physicians. Knowledge and attitudes were measured pre and post intervention.	Post intervention, knowledge scores improved, but there was no change in attitudes. The authors indicate that more dementia training is needed for GPs.
Sass et al. (2019)	United Kingdom (Primary care clinics)	Bespoke distance learning post-graduate certificate addressing dementia assessment, diagnosis, and interventions. Length of graduate certificate program was not reported in the study.	8 GPs	A person-centered dementia education program was conducted at a primary care center with an in-depth case study to evaluate its impact, and barriers and facilitators to implementation. Surveys and self-reports were used.	Knowledge and confidence were improved in participants who perceived the training as positive and helpful. They identified several barriers and reported self-identified improvements in their communication and prescribing practices. Finally, patients and families also reported improved satisfaction.
Thyrian et al. (2016)	Germany (primary care clinics)	This was a sub-group analysis of the ongoing DelpHi-MV randomized controlled trial. The intervention in this trial is a complex intervention with multiple components, the physician education component consists of the GP receiving a detailed report from the patients Dementia Care Manager (DCM) to help guide patient care and treatment decisions. In the current study, both GP’s participating in the DelpHi and non-participating GPs RCT received questions regarding the systematic care of people living with dementia.	257 GPs and nurses	1. Cross-sectional survey of GPs 2. randomized, controlled, prospective intervention DelpHi-MV trial (Dementia: life- and person-centered help in Mecklenburg-Western Pomerania). 3. Intervention group received education about DemTect for early detection, and support through dementia care management (DCM). Attitudes toward dementia assessed via survey.	The majority (89%) of participants found the brief cognitive screening tool (DemTect) helpful. Participants felt DCM was helpful. Interestingly, the authors reported that attitudes were positive in both groups. Authors suggest that this program is feasible, efficacious, well-received, and easily implemented into routine care.
Vancampfort et al. (2023)	Uganda (8 referral hospitals)	One day 8 h workshop that included 5 h of interactive and theoretical sessions and 3 h of practical sessions with role playing exercises and case scenario discussions. Intervention activities focused on 5 core components (1) relevance to participant role and experience, (2) active face to face interaction, (3) underpin practice based learning with theory, (4) delivered by an experienced facilitator, (5) total duration of at least 8 h, (6) support application of learning in practice, and (7) provide a structured tool or guideline to guide care practice.	112 health care professionals, 41 were physicians	Participants completed surveys pre and post education intervention: Alzheimer’s Disease Knowledge Scale (ADKS), Dementia Care Attitude Scale (DCAS), and visual analogue scales (VAS) regarding confidence in specific dementia care skills. Survey completion time was approximately 30 min pre and post intervention. The intervention included 8 h of interactive and practical exercises, including role playing and case studies.	The ADKS, DCAS, and VAS scores improved significantly post-intervention suggesting that the education intervention improved knowledge and attitudes.
Walaszek et al. (2023)	Wisconsin (Primary care clinics)	11 academic detailing visits completed over an 18-month period. Sessions were held in person, virtually, and hybrid during the COVID-19 pandemic. Each visit started with a 30-min didactic presentation on a given topic. Topics were prescribed for the first 5 visits and the last visit, the intervening visit topics were based on clinician needs. Case discussions and in session patient consultations were then held after the didactic session to reinforce learning.	15 clinicians and nurse practitioners	A total of 11 academic detailing visits occurred, utilizing didactic content, case discussions. and in-session patient consultations Clinicians completed surveys at baseline and at 6 and 18 months “to evaluate gains in knowledge, attitudes, and skills for managing BPSD.”	Improvements were observed in knowledge and attitudes about BPSD and the program was well-received. Authors state that academic detail is a feasible and efficacious approach to improving PCPs ability to manage patients with BPSD by improving knowledge, skills, and attitudes.

## 4 Results

**Figure 1:**
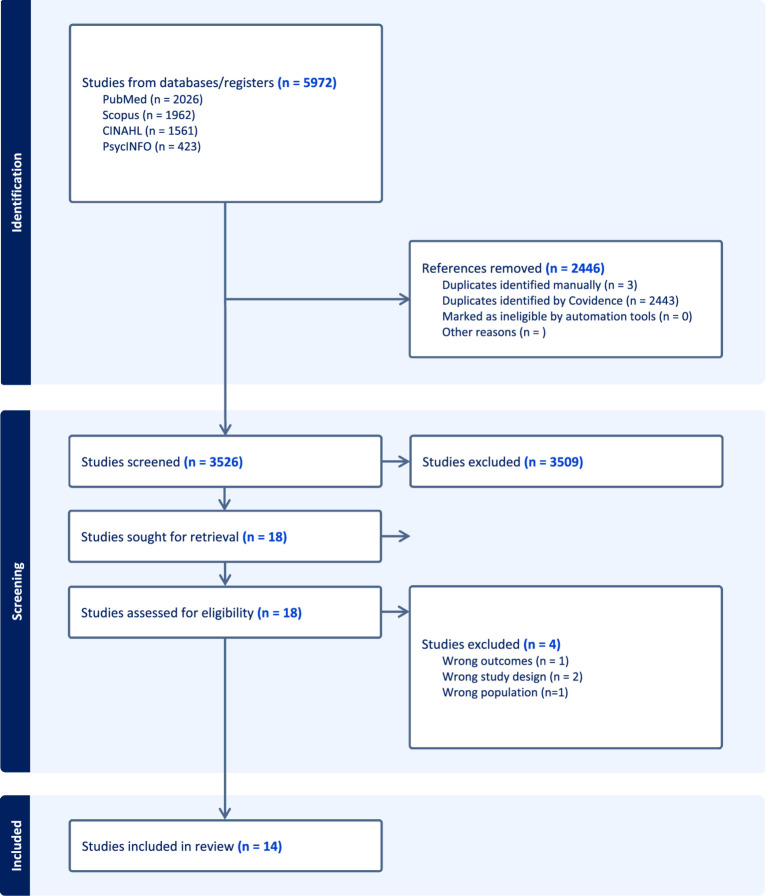
PRISMA diagram.

In total, 5,972 citations were identified and uploaded to Covidence. After removing duplicates, 3,526 titles and abstracts were screened, of which 3,509 were excluded and 18 were assessed for eligibility. A total of four studies were excluded (wrong outcomes, n = 1; wrong study design n = 2; wrong population, n = 1). 14 studies were included in our final analysis (Figure 1).

### 4.1 Descriptive analysis

Of the 14 articles identified, eight were cross-sectional designs with pre- and post-test measures, two were cluster randomized controlled trials, and there were one each of audits, interviews, & questions, case study, mixed methods, and quasi-experimental design. Four studies were conducted in Australia (Bentley et al., 2019; Crombie et al., 2024; Pond et al., 2018); three were conducted in the United States (Perales-Puchalt et al., 2022; Walaszek et al., 2023; Albrecht et al., 2022); two studies were conducted in the United Kingdom(Edwards et al., 2015; Sass et al., 2019); one was conducted in Canada (Arsenault-Lapierre et al., 2022), Germany (Thyrian et al., 2016), Brazil (Sá Mayoral et al., 2021), and Uganda (Vancampfort et al., 2023). One study (Cartz-Piver et al., 2023) spanned multiple countries (France, Bulgaria, and Poland).

### 4.2 Interventions

The reviewed studies predominantly employed interventional designs aimed at addressing stigma, improving confidence, and enhancing knowledge among physicians about the complexities of dementia, including elements of both biomedical information and stigma-related misconceptions and negative attitudes. Of the studies analyzed, two were RCTs (Pond et al., 2018; Thyrian et al., 2016), five studies adopted pilot study frameworks to evaluate the feasibility and initial impact of interventions (Albrecht et al., 2022; Cartz-Piver et al., 2023; Crombie et al., 2024; Edwards et al., 2015; Sass et al., 2019). Two were cluster randomized trials (Mason et al., 2020; Pond et al., 2018), and one was mixed-methods (Crombie et al., 2024). All studies found statistically significant improvements in at least one outcome measure, however one study only identified a significant improvement after secondary analysis (Pond et al., 2018).

The interventions utilized a range of formats, targeting both stigma and/or negative attitude reduction and the practical skills necessary for dementia care. No studies were identified that used social contact or experiential learning. Educational workshops/training programs that lasted a single day or less were the most common intervention type, employed in eight studies, (Albrecht et al., 2022; Bentley et al., 2019; Cartz-Piver et al., 2023; Edwards et al., 2015; Mason et al., 2020; Perales-Puchalt et al., 2022; Sá Mayoral et al., 2021; Vancampfort et al., 2023), to enhance knowledge, confidence, and attitudes toward dementia care. Four studies integrated person-centered frameworks, (Albrecht et al., 2022; Edwards et al., 2015; Sass et al., 2019; Thyrian et al., 2016), which emphasizes holistic care practices and interdisciplinary collaboration. Academic detailing, which combines didactic lectures, case discussions, and patient consultations, was used in two studies (Walaszek et al., 2023 & Pond et al., 2018). Two studies, (Albrecht et al., 2022; Bentley et al., 2019), implemented online modular training to address time constraints and access concerns for physicians. Albrecht et al. (2022) used a combination of online (during the COVID-19 pandemic) and in-person trainings that employed the DICE framework, a structured tool specifically targeting the management of behavioral and psychological symptoms of dementia (BPSD) (e.g., apathy, aggression, and depression, etc.). The intervention by Vancampfort et al. (2023) also included BPSD management in their training protocol, but in a broader educational context including communication, knowledge, and attitudes. Only one intervention included a specific focus on recent advances in Alzheimer’s research and how to integrate that research into everyday care (Perales-Puchalt et al., 2022).

### 4.3 Intervention types

A wide range of educational and skill-building were identified in this review. Cartz-Piver et al. (2023) implemented an anti-stigma intervention that integrated ethical reasoning and practical approaches alongside traditional academic content. This program demonstrated significant improvements in reducing negative stereotypes, stigma, communication difficulties, and perceptions of diagnostic futility among participants. Importantly, clinical confidence increased across all scenarios, with younger participants and those with less dementia-related experience benefiting the most (Cartz-Piver et al., 2023). These findings highlight the importance of incorporating ethical and practical dimensions into training programs to empower GPs to address dementia care confidently and effectively across their career span.

Other studies also focused on building confidence and reducing stigma. Crombie et al. (2024) observed improvements in confidence and attitudes following a pilot training program for GPs and practice nurses. Prior to the intervention, many GPs expressed negative attitudes and nihilistic views of dementia diagnosis, reporting that it was easier to give a diagnosis of cancer rather than dementia. They also noted the difficulty in giving a dementia diagnosis due to limited treatment options and a belief that patients may not want to know if they have dementia. Post-intervention, GPs expressed more positive attitudes, took a more active role in screening, and reported increased confidence in conversations with patients and families about dementia. Attitudes positively improved from 30% agreement pre-training to 79% post-training, and improvements were observed in knowledge, confidence in dementia management, and confidence in diagnosis disclosure (median increase 2.5/10, 3/10, and 2.75/10, respectively) (Crombie et al., 2024).

BPSD often presents a challenge to providers and caregivers alike, the management of which is a critical area of dementia care whereby stigma and lack of education often present barriers (Warren, 2023b). Academic detailing is an interactive educational approach in which trained health professionals provide tailored information to clinicians to persuade clinicians to change clinical approaches to improve best practices (Walaszek et al., 2023). Walaszek et al. (2023) implemented an academic detailing approach that included didactic lectures, case discussions, and patient consultations. Their intervention improved physician knowledge and attitudes toward managing BPSD and increased satisfaction with training. Albrecht et al. (2022) employed the DICE (Describe, Investigate, Create, Evaluate) approach to train dementia care professionals, demonstrating significant improvements in knowledge, attitudes, and self-efficacy in managing BPSD. These interventions showed that practical, structured approaches can empower physicians to address complex behavioral challenges in dementia care effectively.

In the general practice setting, the evidence-based approach of academic detailing provides clinically relevant information while also addressing the needs and concerns of clinicians (Pond et al., 2018), including the management of BPSD (Walaszek et al., 2023). Walaszek et al. (2023) showed that this approach, involving personalized case discussions and consultations, increased physician confidence and attitudes towards persons with dementia who have BPSD, with high satisfaction with the program. Pond et al. (2018) also used academic detailing in a cluster randomized trial, improving patient satisfaction and caregiver enablement, although primary outcomes related to dementia care quality showed no significant improvement.

Edwards et al. (2015) designed an educational intervention emphasizing person-centered dementia care and the importance of team-based efforts within primary care settings. This program led to significant improvements in understanding person-centered practices, recognizing non-cognitive dementia symptoms, and appreciating the value of non-clinical staff in dementia recognition. Similarly, Sass et al. (2019) implemented a collaborative interdisciplinary person-centered training program, resulting in improvements in communication and prescribing practices, as well as increased satisfaction among patients and families.

Given the time-constraints faced by primary care providers (PCPs) (Arsenault-Lapierre et al., 2022), brevity is an asset in continuing medical education. Time-efficient educational interventions have also shown promise in this regard. For example, Bentley et al. (2019) developed an online resource with modular training designed to improve knowledge, confidence, and attitudes in just 3 h. This program led to measurable improvements in dementia-related skills and clinical behaviors, offering a practical solution for time-constrained physicians. However, it’s important to note that research by Sá Mayoral et al. (2021) revealed that even brief, structured educational programs may fail to shift deeply entrenched attitudes, such as nihilistic views of dementia care. This underscores the need for experiential learning components, including direct engagement with patients, to dispel stigma more effectively (Sá Mayoral et al., 2021).

Cultural and contextual factors also play an essential role in shaping the success of dementia education interventions. In low-income countries, Vancampfort et al. (2023) explored the feasibility and efficacy of brief educational interventions tailored to resource-limited settings. Their program, delivered to physicians and healthcare professionals in Uganda, significantly improved dementia-related knowledge, attitudes, and confidence. In a similar vein, Crombie et al. (2024) sought to evaluate GPs competency, confidence, and attitudes toward dementia in the rural and urban contexts (1 urban clinic serving a primarily black community and 1 rural center serving a primarily white community) and found that a targeted training program demonstrated improvements across knowledge, attitudes, and confidence measures that were previously lacking.

### 4.4 Outcome measures

A variety of outcome measures were used across the studies to measure knowledge, confidence, attitudes, and stigma. Surveys and questionnaires that utilized Likert scales or visual analogue scales (VAS) were used for acceptability and satisfaction about the intervention(s). Specific measures for stigma, attitudes, confidence, and knowledge will be discussed briefly as follows.

### 4.5 Stigma

Several studies employed tools specifically designed to evaluate stigma among healthcare providers. The Dementia Negative Stereotype Scale (DNS), used by Cartz-Piver et al. (2023), quantified changes in negative stereotypes about dementia. This instrument revealed small but statistically significant reductions in stigma post-intervention, with scores decreasing from 38.7 to 35.5%. The DNS proved particularly effective in capturing shifts in specific biases and negative perceptions, offering valuable insights into the intervention’s impact on GPs.

### 4.6 Evaluating confidence and attitudes

Tools measuring confidence and attitudes were instrumental in understanding the potential for stigma reduction of educational interventions. The Dementia Clinical Confidence Scale (D-CO), a 16-item scale with Likert rating, also used by Cartz-Piver et al. (2023), assessed clinical confidence in dementia care across different clinical situations. Post-intervention, the scale showed significant improvements across all clinical scenarios, reflecting increased self-efficacy among participants.

The General Practitioners Confidence and Attitude Scale for Dementia (GPACS-D) was employed across multiple studies, including Mason et al. (2020), Walaszek et al. (2023), Perales-Puchalt et al. (2022), and Bentley et al. (2019). This scale contains three sub-scales measuring confidence in clinical abilities (six items), attitude to care (six items), and engagement (three items), with Likert ratings for each item. It can also have tailored adaptations for specific contexts such as BPSD in the study by Walaszek et al. (2023). Across these studies, the GPACS-D consistently reflected significant improvements in all sub-scales, demonstrating its potential reliability as a tool for evaluating educational outcomes.

The Dementia Care Attitude Scale (DCAS), used by Vancampfort et al. (2023), is a 10-item Likert rating scale ranging in scores from 10 (most negative attitude) to 50 (most positive attitude). It provided a comprehensive measure of healthcare professionals’ attitudes toward dementia care. Significant improvements (37.3 to 41.8) were observed following the intervention.

### 4.7 Assessing knowledge and educational impact

As most interventions included a competency component, instruments assessing knowledge gains and educational impacts were a fundamental component of training programs. The Knowledge About Memory Loss and Care (KAML-C), a 9-item scale scored by the number of correct answers, used by Albrecht et al. (2022), demonstrated a small but technically statistically significant post-intervention improvements in participants’ understanding of dementia care and BPSD management (7.9 to 8.0, *p* = 0.04). This instrument effectively captured the educational impact of the DICE framework in this regard.

Similarly, the Dementia Knowledge Assessment Scale (DKAS), a 25-item scale with factually correct and incorrect answers for each item scored out of 50 points, was applied by Bentley et al. (2019). It measured increases in dementia awareness and clinical knowledge following a brief online training program for international medical graduates (39.7 to 43.7). Its focus on knowledge acquisition makes it well-suited for interventions emphasizing accessible and efficient educational delivery.

The Alzheimer’s Disease Knowledge Scale (ADKS), which comprises 30 true/false statements about dementia covering seven domains (life impact, risk factors, treatment & management, assessment and diagnosis, caregiving, symptoms, and disease course) was used by Vancampfort et al. (2023) in association with their brief intervention in Uganda. The ADKS was measured before and after a 1-day, 8-h training session for physicians practicing in a low-income country, and results represented improvements in dementia knowledge post-training (19.0 to 22.8).

The Knowledge and Attitudes Quiz about Dementia, used by Sá Mayoral et al. (2021) contains 14 multiple choice questions to assess knowledge with three sub-scales: epidemiology (3 questions), diagnosis (8 questions), and management (3 questions). The Attitude Quiz contains 10 sentences about physicians’ thoughts on managing patients with dementia, with each sentence having an associated Likert scale. The instrument utilized by Sá Mayoral et al. (2021) was specifically adapted for a Brazilian context and assessed both knowledge and attitudes pre- and post-intervention. While improvements in knowledge were observed (8.35 correct answers to 9.97 correct answers), attitudes remained largely unchanged, highlighting the challenge of addressing deeply entrenched stigma through brief training.

### 4.8 Behavioral and outcome-oriented measures

Several studies incorporated tools that linked physician changes to real-world outcomes. Kirkpatrick’s Evaluative Framework, used by Sass et al. (2019) as part of an academic detailing intervention, assessed reactions, learning, behavior changes, and outcomes. Participants reported improvements in communication practices and prescribing behaviors, as well as increased satisfaction among patients and families, reflecting the framework’s ability to capture the broader impact of educational interventions.

The Visual Analogue Scales (VAS), which is simply a scale that participants can move between 0 and 100 to indicate agreement with a given item or confidence in a skill, was applied by Vancampfort et al. (2023), and offered a straightforward method for assessing confidence across specific dementia care skills. Its adaptability to low-resource settings demonstrated its utility in contexts with limited resources. Studies such as Arsenault-Lapierre et al. (2022), Crombie et al. (2024), Edwards et al. (2015), Thyrian et al. (2016), and Pond et al. (2018) utilized surveys and questionnaires that combined assessments of knowledge and competencies, attitudes, and confidence. Further, Pond et al. (2018) also utilized secondary measures that indirectly reflected physician attitudes and behaviors. These included patient satisfaction, caregiver enablement, and quality of communication. While not specifically designed to measure stigma, these outcomes provided a holistic view of the interventions’ effects on care delivery and captured attitudinal components reflective of stigma and bias.

In summary, the instruments used across these studies captured a range of outcomes, from stigma reduction and confidence building to knowledge enhancement and practical application in dementia care. Tools like the DNS, OMS-HC, and GPACS-D were particularly valuable in measuring shifts in stigma and attitudes, while the DKAS and KAML-C provided insights into the educational impacts of the interventions. Frameworks such as Kirkpatrick’s evaluative model and patient-centric measures added depth by linking physician changes to tangible improvements in patient and caregiver experiences.

## 5 Discussion

Dementia-related stigma is a well-documented problem that reduces quality of life in persons with dementia and caregivers (Alzheimer’s Disease International, 2019; Bacsu et al., 2022), yet the current state of the literature on stigma-reducing interventions is largely unexplored (Bacsu et al., 2022), including in physicians. Reducing dementia-related stigma may lead to improved access to care, utilization of support resources, and improved quality of life in persons with dementia and their families (Herrmann et al., 2018). Furthermore, the body of research addressing physician stigma in dementia care highlights the significant barriers it poses to timely diagnosis and optimal treatment, along with the potential strategies to mitigate these challenges. Stigma manifests as negative attitudes about persons with dementia and nihilistic attitudes about their prognoses among physicians, negatively affecting patient care and outcomes (Sá Mayoral et al., 2021). Research by Cartz-Piver et al. (2023) demonstrated that general practitioners (GPs) often experience uncertainty about their roles in dementia care, fears about imposing stigma through a diagnosis, doubts about the benefits of early diagnosis, and challenges in communication. These findings are consistent with other studies indicating that stigma not only hinders effective diagnosis but also undermines effective care by exacerbating delays in care and future planning for patients and caregivers (Bacsu et al., 2022). Interventions aimed at reducing stigma, improving confidence (e.g., Cartz-Piver et al., 2023), and equipping physicians to manage BPSD (e.g., Albrecht et al., 2022; Walaszek et al., 2023) have provided valuable insights into effective approaches. The persistence of these attitudes underscores the critical need for targeted interventions to address stigma among healthcare providers. Therefore, the aim of this scoping review was to synthesize the existing literature to identify key features of stigma-reducing interventions targeted to physicians.

To categorize the interventions, we were guided by two main frameworks. Informed by Corrigan and Penn’s (1999) framework of stigma reduction approaches (protest, education, contact), we observed that education was commonly prioritized in these studies, while contact and protest were not included. Of note, it has been postulated that the unilateral use of protest may result in a rebound effect that exacerbates stigma (Corrigan and Penn, 1999). Further informed by the theory of planned behavior (Ajzen, 1991) which explains behavior as a product of behavioral, normative, and control beliefs that give rise to the broader constructs of attitudes, intentions, and self-efficacy (Ajzen, 1991; Bosnjak et al., 2020), we observed similarities in the common theme of addressing knowledge, skills, attitudes, confidence, and behavior to decrease stigma. A variety of educational approaches were utilized. In addition to factual content, key components appeared to utilize ethics; processes of diagnosis and management (including screening, symptom recognition, and BPSD); physician’s emotional involvement including feelings of anxiety and helplessness; communication strategies; prescribing practices; carer stress; legal, end of life issues and advanced directives; support services and respite; referral to specialists; caregiver involvement; person-centered approaches; and biopsychosocial approaches. No studies utilized direct patient contact, and few addressed the importance of cultural and geographical contexts (e.g., see Vancampfort et al., 2023; Walaszek et al., 2023). These interventions addressed stigma directly or indirectly using a combination of knowledge, confidence, and attitudes in physicians.

The extant literature on addressing stigma in dementia care has generated valuable insights and significantly advanced our understanding of the barriers faced by physicians and potential strategies for improving their attitudes and behaviors. Key educational interventions that include components of skill-building, confidence and attitudes, ethical and person-centered approaches, interdisciplinary training, and time-efficient approaches, have demonstrated some preliminary efficacy in reducing stigma, enhancing confidence, and equipping physicians to manage complex challenges such as BPSD. However, critical gaps persist in understanding the long-term sustainability of these interventions, the causal mechanisms driving change, and the optimal methods for tailoring approaches to diverse physician demographics, cultural contexts, and practice locations. Future efforts must prioritize longitudinal randomized studies, experiential learning, standardized measurement tools, and global inclusivity to address these limitations. Furthermore, there are additional barriers faced by physicians that must be addressed to holistically approach dementia stigma reduction. For example, physicians often work in high-pressure environments with institutional time constraints, creating more difficulty for comprehensive dementia assessments that require longer consultations (Bacsu et al., 2020). These time constraints remain a significant barrier to quality of care in general, and also to physician participation in dementia-related training. Many providers operate under high patient loads and productivity demands, which limits opportunities for extended education sessions. Therefore, stigma-reduction interventions must be designed with flexibility in mind, favoring brief, modular formats that can be integrated into existing clinical workflows or accessed asynchronously. This approach increases feasibility and uptake without sacrificing impact. Additionally, we would be remiss not to mention the larger institutional structures that perpetuate this barrier. Notwithstanding, by building on these insights, the field can foster a stigma-free, person-centered healthcare environment that enhances outcomes for individuals living with dementia and their families, ensuring more effective and inclusive dementia care practices worldwide. Future research should address several key questions: (1) Which specific components, or combination of components, of provider training are most effective in reducing stigma toward dementia? (2) How do changes in provider attitudes translate into measurable behavioral changes in clinical settings? (3) What role do factors such as provider discipline, years of experience, and exposure to people living with dementia play in shaping stigma reduction outcomes? Addressing these questions would allow for a more nuanced understanding of intervention efficacy and implementation needs.

### 5.1 Stakeholder considerations

While the primary stakeholder is the physician in physician-related stigma of dementia, everything in the periphery ultimately affects the patient. Caregivers and the caregiver-patient dyad are also affected by physician stigma, and have demonstrated benefit by its reduction, as evidenced by the feedback elicited from the dyad by Pond et al. (2018) who reported improved empathy and communication after the training to improve attitudes and confidence in physicians.

By developing evidence-based interventions to address negative attitudes, stigma, and lack of sufficient dementia education (including BPSD management) in physicians, researchers can support physicians and caregivers in these areas to contribute to better patient care (Albrecht et al., 2022; Walaszek et al., 2023), and physicians can inform researchers and educators about their needs (Arsenault-Lapierre et al., 2022). Healthcare administration and other healthcare personnel may likewise exacerbate or reduce stigma via organizational procedures and time allocation, the amount of support from nurses and staff, and having stigmatizing attitudes themselves. The content of dementia and geriatric education varies considerably among medical schools (Bacsu et al., 2020), and in this way may further help or hinder levels of education, confidence, and attitudes towards persons with dementia. Medical education can also facilitate the improvement of attitudes and dementia care management in the personnel who support the physician (Crombie et al., 2024; Thyrian et al., 2016). These educational efforts also have downstream effects on patients and caregivers.

### 5.2 Assessment of knowledge gaps

Despite these advancements, several knowledge gaps persist in the field. One of the most salient is the lack of evidence on the long-term impact and sustainability of educational interventions. While many studies reported rapid improvements in attitudes, knowledge, and confidence, there is limited understanding of whether these changes endure over time or translate into sustained behavior changes in clinical practice. Future research should employ longitudinal designs to track the durability of intervention outcomes and their impact on patient care.

Additionally, the causal mechanisms linking stigma reduction to improved clinical outcomes remain unclear. Although studies suggest that increased confidence and reduced stigma correlate with better care practices, the precise pathways driving these changes have not been fully elucidated. Understanding these mechanisms may better inform the optimal designs for interventions that effectively target the root causes of stigma.

The variability in intervention efficacy across different physician demographics is another area requiring further exploration. Gender, racial, and cultural differences in attitudes toward aging and elder care both reflect and influence attitudes and behavior (Low and Purwaningrum, 2020). The role these factors may play in physician attitudes towards caring for persons with dementia is poorly understood and requires further exploration. Further, evidence suggests that having direct patient contact with persons with dementia is correlated with more positive attitudes and greater knowledge (Scott et al., 2019). The literature suggests that ethnicity and culture are factors associated with stigma, and that negative attitudes and stigma may be more pronounced in those who have limited knowledge and/or contact with persons with dementia, males, and younger individuals (Harper et al., 2018; Herrmann et al., 2018). The extent to which these factors translate to physicians is still unknown but underscores the need for tailored interventions that address the specific barriers faced by different groups of healthcare providers.

The inclusion of persons with dementia in educational programs represents an important but underutilized strategy (Treadaway et al., 2019). As Sá Mayoral et al. (2021) noted, direct interactions with individuals living with dementia can provide experiential learning opportunities that challenge negative stereotypes and promote empathy. Including experiential learning components, such as direct interactions with persons with dementia and their caregivers, could enhance empathy and challenge stereotypes more effectively. Expanding this approach could enhance the applicability of stigma reduction interventions.

Integration of BPSD education and management into routine practice is an additional gap in research and applied care practices. While structured frameworks like DICE and academic detailing have shown promise, their integration into routine care remains limited. Future research should evaluate the systemic implementation of these frameworks and their impact on patient and caregiver outcomes.

Another significant gap lies in the integration of person-centered and interdisciplinary care models into routine practice. While these approaches have demonstrated success in improving attitudes and communication, their systemic implementation remains limited. Future research should evaluate the feasibility and impact of integrating such models into standard care practices, particularly in resource-constrained settings.

While not lengthy, there are a variety of measures used to evaluate negative attitudes and stigma in physicians. Lack of consistency in instruments used pose a challenge to synthesizing findings across studies. Variability in how stigma, attitudes, and confidence are measured makes it difficult to compare results or draw generalizable conclusions. We recommend comparing these measurements to compare validity and reliability to determine if there is a “best available” or if a combination approach would be most useful to utilize. Collectively, these instruments may inform a comprehensive evaluation strategy, though future standardization efforts could enhance comparability across studies and further strengthen the field. Additionally, a majority of the measures utilized in the identified studies consisted of either answering questions or offering self-reports on changes in a given outcome. Evidence has shown that passing a test does correlate strongly with future clinical performance (i.e., knowing how to answer a question on a test and correctly handling a scenario in real life are different things) (Shirkhodaie et al., 2023). Furthermore, self-reported measures are notoriously unreliable due to factors such as social desirability bias (Fisher and Katz, 2000), and would be more contextualized by triangulation of data. Determining the construct and content validity of these instruments, assessing their applicability across diverse demographics, and creating unified scales that can be used across studies to evaluate different constructs would enhance the comparability, reliability, and generalizability of future research.

Finally, cultural sensitivity and global applicability are further concerns. Interventions in high-income countries dominate the field, with fewer studies addressing dementia care in culturally diverse or low-resource settings. Expanding research to include culturally specific approaches and diverse healthcare contexts is essential for developing globally applicable solutions.

### 5.3 Next steps for continued exploration

Addressing the identified barriers while leveraging existing facilitators is essential to advancing knowledge and practice in dementia care. Future research must prioritize longitudinal studies to evaluate the sustainability of interventions and randomized studies to isolate the effects of different interventions and better understand the mechanisms underpinning their induced changes. Many of the evaluated studies in this review had short follow-up periods, understanding whether initial improvements in stigma, confidence, and knowledge persist over time will provide critical insights into the long-term impact of educational programs.

Expanding culturally and contextually tailored interventions, including in low- and middle-income countries is also imperative. Efforts should include collaboration with local stakeholders to adapt educational content and delivery methods to the needs of specific populations. This approach has been shown to improve both acceptance and effectiveness in diverse settings, as evidenced by Vancampfort et al. (2023).

To combat entrenched stigma, researchers should incorporate experiential learning components into training programs. Direct interactions with individuals living with dementia and their caregivers could challenge deeply rooted biases that persist despite traditional education methods. Simultaneously, there is a need to streamline systemic integration of dementia care management systems (DCM) into primary care workflows. As highlighted by Thyrian et al. (2016), such systems can facilitate earlier detection and foster interdisciplinary collaboration.

Building on the success of brief interventions, future efforts should scale modular training formats to make education more accessible globally. Modular training was identified as a potentially useful strategy, especially given its adaptability across provider disciplines and settings. To enhance its effectiveness, content should be structured around core domains identified in the review: (1) foundational knowledge of dementia and its subtypes; (2) stigma awareness and its manifestations in clinical care; (3) person-centered communication strategies; (4) real-world case simulations and role-play; and (5) reflective practice modules. Modules should be adaptable to different disciplines (e.g., primary care, nursing, neurology) and include flexible formats such as asynchronous online learning, brief in-service workshops, and interactive e-learning platforms. Including testimonials from people living with dementia and their caregivers can also enhance empathy and engagement by adding the component of lived experience. Combining these approaches with standardized outcome measures, such as the GPACS-D and OMS-HC, will enhance comparability across studies and support the development of evidence-based best practices.

Finally, technological innovation holds promise for expanding the reach and efficiency of dementia care education. Tele-education platforms and decision-support tools could provide practical solutions for clinicians managing dementia in resource-limited environments. Researchers should also explore the emotional and behavioral dimensions of stigma, such as social distancing and feelings of helplessness, to develop interventions that target these nuanced barriers (Beaulieu et al., 2017). Another potential avenue for technological implementation is the use of virtual reality (VR). While the studies have not been conducted in physicians to the best of our knowledge, recent evidence is suggesting that VR interventions may reduce implicit bias and improve attitudes towards persons with dementia (Matsumoto et al., 2024).

### 5.4 Limitations

This review aimed to summarize the extant literature to identify dementia stigma-reduction interventions targeted to physicians that may inform key stakeholders aiming to improve the quality of care for persons with dementia and their caregivers. However, it is not without limitations. This review was restricted to manuscripts in English that were available in full text through our university’s library platform and did not include grey literature. Literature prior to 2010 was also excluded. As a result, it is possible that pertinent research was missed. Additionally, while the restriction of our search criteria to only interventions meant for physicians was necessary given our research question, it also inherently limited the breadth of interventions we could evaluate. A more comprehensive search may reveal interventions that have proven effective in reducing dementia stigma among other health care workers and which may work for physicians as well. A great amount of primary care practices involve nurse practitioners and physician assistants, who may be the first line of contact before the PCP, which is a further limitation of our manuscript and an opportunity for future research. As previously stated, although this review focused primarily on interventions targeting physicians, it is important to acknowledge that non-physician primary care providers, such as nurse practitioners (NPs) and physician assistants (PAs), are increasingly central to dementia care delivery. Recent data show that from 2013 to 2019, the proportion of Medicare visits provided by NPs and PAs in the U.S. rose from 14 to 25.6% (Patel et al., 2023). Despite this trend, there remains a lack of targeted stigma-reduction interventions and research specific to these provider groups. While one study included NPs in its sample (Walaszek et al., 2023), the majority of interventions in this review were not explicitly designed to address the unique roles or practice contexts of NPs and PAs. This represents an important gap and opportunity for future research to develop tailored interventions for this rapidly expanding segment of the dementia care workforce. Further, as inherent to the scoping review process, the articles included in this review were not assessed for quality or bias. Finally, further research is needed to establish the mechanisms and long-term effects of the interventions in practice, if any.

## 6 Conclusion

Dementia-related stigma in providers is associated with a lack of education, misperceptions, and biases (Alzheimer’s Disease International, 2019). The content of dementia and geriatric education varies considerably among medical schools (Bacsu et al., 2020), but research suggests that didactic and patient-exposure interventions can increase knowledge, skills, and empathy in medical students (Goldman and Trommer, 2019), highlighting the critical need to better identify and measure stigma and novel approaches to stigma-reduction interventions (Herrmann et al., 2018).

Given the rapid rate at which the population is aging, PCPs will become even more central to, and involved in, dementia care management (Walaszek et al., 2023). By addressing existing barriers and building on identified facilitators, the field can advance toward creating a more inclusive and effective system for dementia care education. Improvements in research, training, and systematic reform are crucial next steps for fostering sustainable change. Through these efforts, healthcare systems can better equip practitioners to provide stigma-free, person-centered care that improves outcomes for individuals living with dementia and their families.

## Data Availability

The original contributions presented in the study are included in the article/supplementary material, further inquiries can be directed to the corresponding author.
