# Correction: Dementia-related stigma in physicians: a scoping review of stigma-reduction interventions

**DOI:** 10.3389/frdem.2025.1678458

**Published:** 2025-09-29

**Authors:** Alison Warren, Zan Wynia

**Affiliations:** ^1^The Department of Clinical Research and Leadership, School of Medicine and Health Sciences, George Washington University, Washington, DC, United States; ^2^The Frame-Corr Laboratory, The George Washington University School of Medicine and Health Sciences, Washington, DC, United States; ^3^Harvard University Extension School, Division of Continuing Education, Cambridge, MA, United States; ^4^Anschutz Medical Campus General Internal Medicine, University of Colorado, Aurora, CO, United States

**Keywords:** stigma, bias, dementia, Alzheimer's disease, intervention, physician, healthcare personnel

In the published article, a PRISMA diagram was erroneously omitted. The PRISMA diagram should have been placed after Section 4 (**Results**) and before Section 4.1 (*Descriptive Analysis*) in the article. This Figure 1 has been included below.

**Figure 1:**
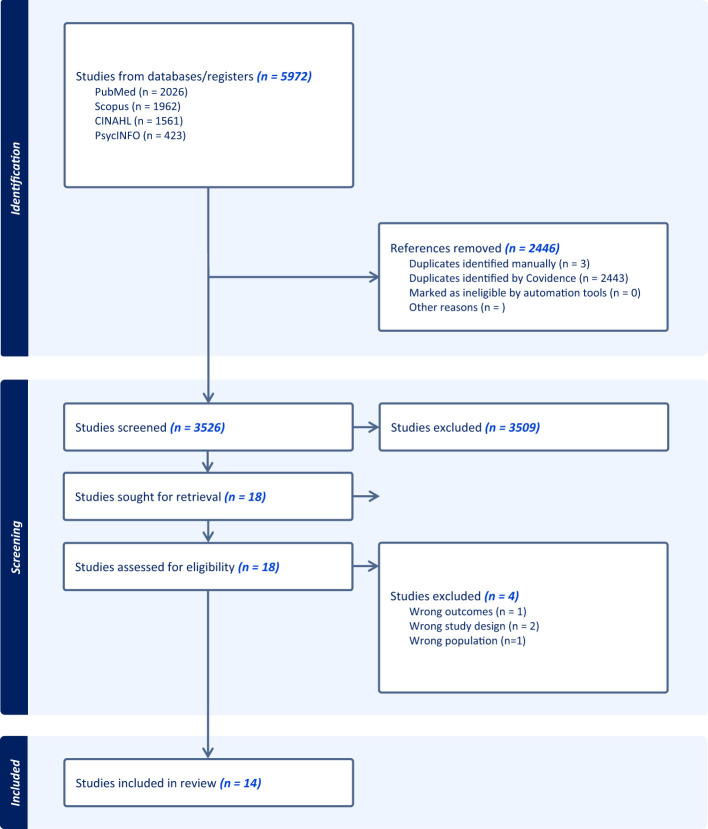
PRISMA diagram.

The original version of this article has been updated.

